# Supplementary material for: Resurgence of Clinical Malaria in Ethiopia and Its Link to Anopheles stephensi Invasion
Source: Pathogens. 2024 Aug 31;13(9):748. doi: 10.3390/pathogens13090748 (PMC11435327; doi:10.3390/pathogens13090748)
Supplement: Supplementary file 1 [file pathogens-13-00748-s001.zip › pathogens-3159349-supplementary.pdf]

Supplement

Figure S1. Map of the malaria outbreaks detected at the 33 selected sites in Ethiopia in 2022 and *An. stephensi* presence status at each site

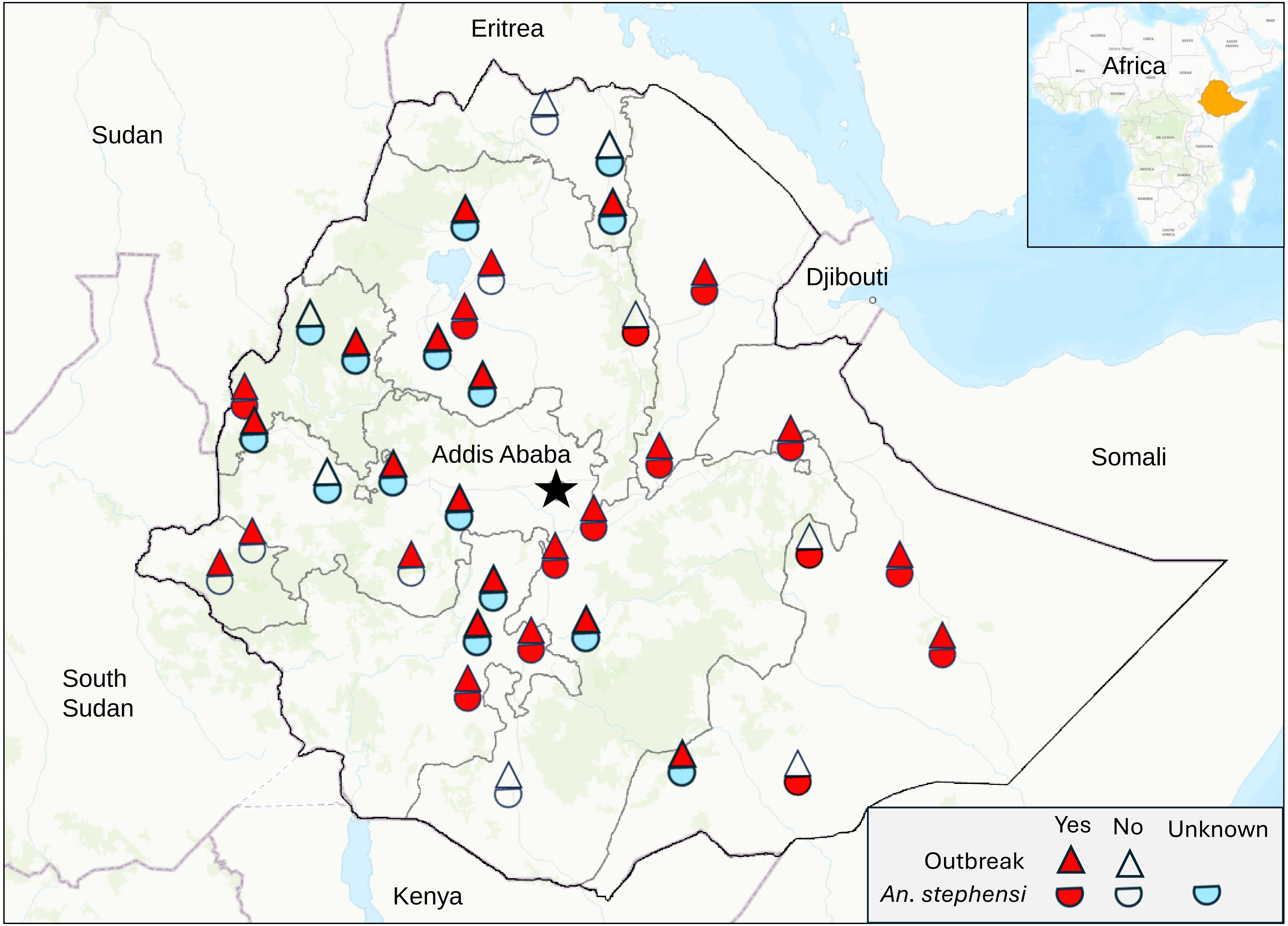

Figure S2. Dynamics of clinical malaria cases from 2013 to 2022. Sites with low case numbers but with sudden increase in clinical malaria cases recently

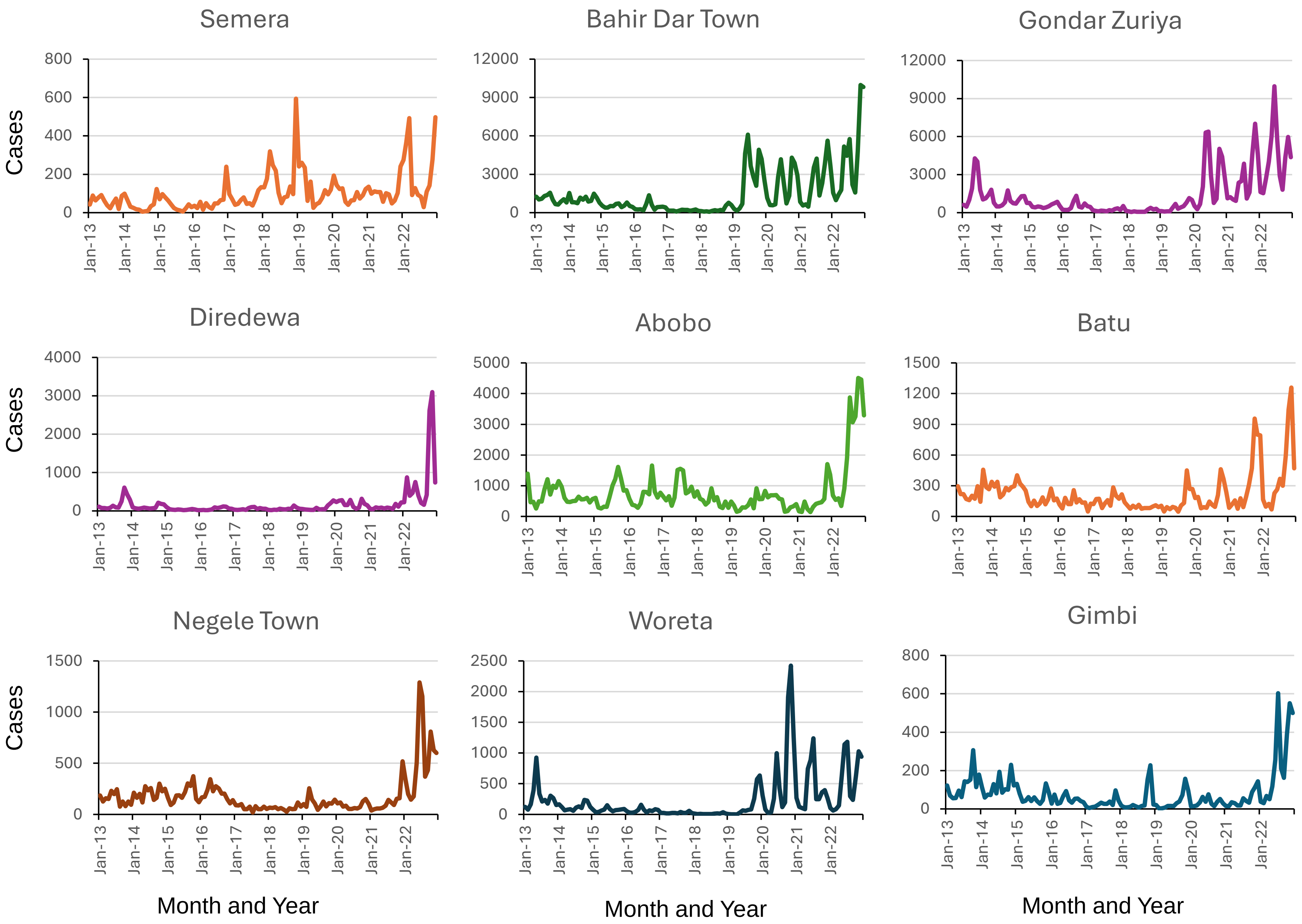

Figure S3. Dynamics of clinical malaria cases from 2013 to 2022. Sites with declining case numbers but with sudden increase in clinical malaria cases recently

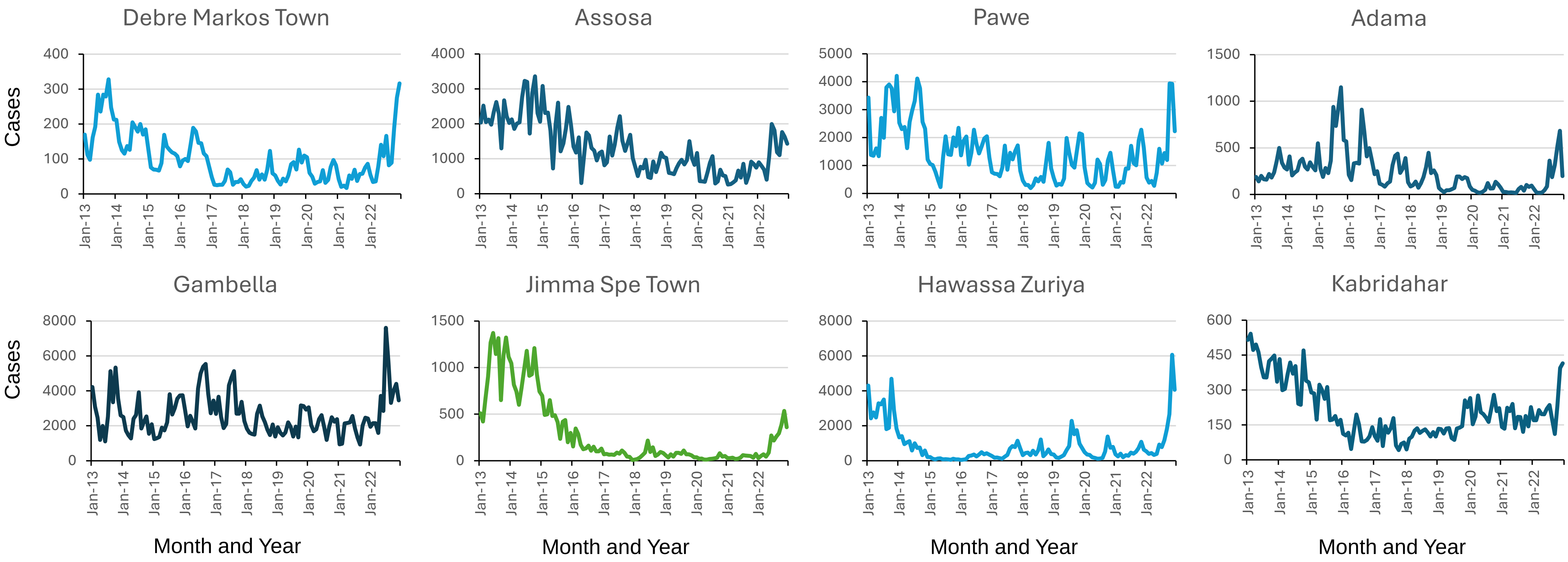

Figure S4. Dynamics of clinical malaria cases from 2013 to 2022. Sites with and no recent increase in clinical malaria cases

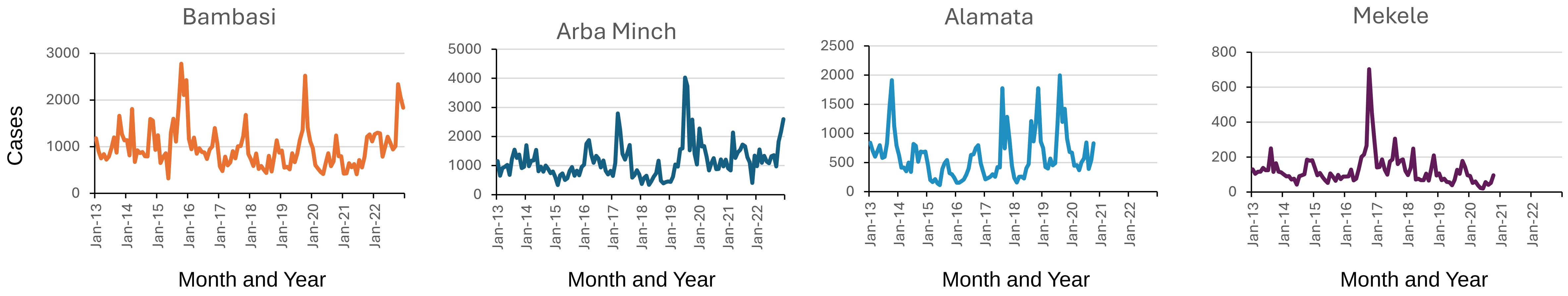

Figure S5. Dynamics of clinical malaria cases from 2013 to 2022. Sites with continuous declining trend in clinical malaria cases

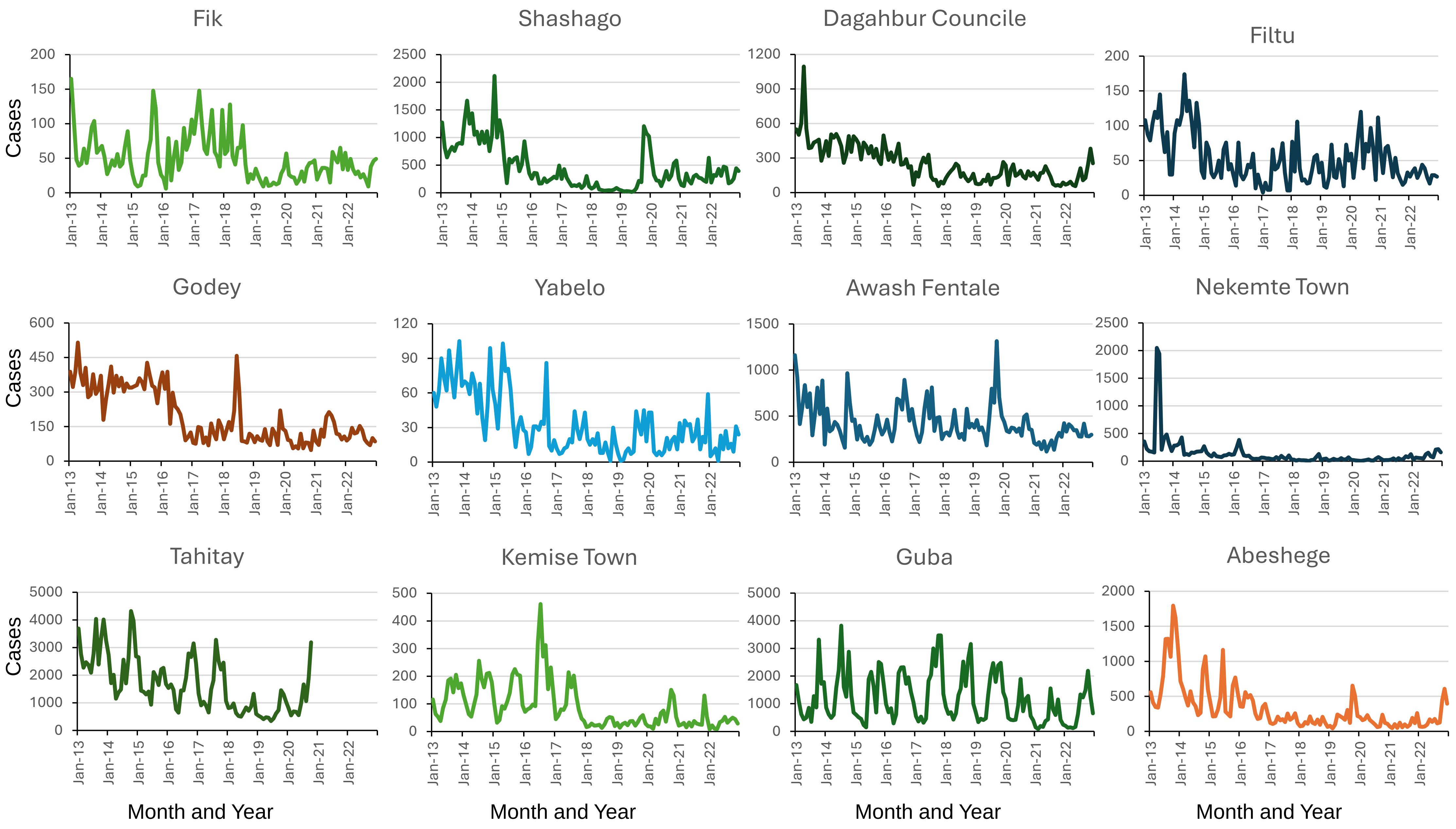

Supplement

Table S1. Average changes in clinical malaria incidence from 2017 to 2022 by risk levels defined by observed API in 2017

2017 risk level	Average changes in malaria incidence (range)					
	Increased		No change		Reduced	
	Mean (range)		Mean (range)		Mean (range)	
API = 0	0.9	(0, 6.9)	0.0	(0, 0)	NA	
API ≤ 5	28.1	(0.8, 290.5)	0.5	(-3.4, 3.9)	-0.1	(-0.1, -0.1)
5 < API < 10	34.3	(1.4, 201.2)	0.2	(-3.6, 4.0)	-4.0	(-8.4, -0.5)
10 ≤ API < 50	97.4	(15.2, 462.1)	7.7	(-32.5, 39.1)	-12.5	(-38.4, -1.1)
API ≥ 50	NA		20.3	(-253.5, 694.9)	-92.5	(-776.9, -7.4)

API: annual parasite incidence. NA: not applicable. No change, reduced and increased represent risk levels (based on API categories) did not change, reduced and increased from 2017 to 2022, respectively, based on API observations in 2017 and 2022.

Supplement

Table S2. Malaria outbreak detection and *An. stephensi* status and proportion in selected sites

Region		Outbreak detection			*Anopheles stephensi* §	
	Woreda	Status †	Rate ratio ‡	P-value	Status	Proportion
Afar	Awash Fentale	No	0.87	0.0573	Yes	92%
	Semera	Yes	1.83	0.0334	Yes	100%
Amhara	Bahir Dar Town	Yes	2.69	0.0088	No	0%
	Debre Markos Town	Yes	2.46	0.0069	No	0%
	Gondar Zuriya	Yes	3.41	0.0007	No	0%
	Kemise Town	No	0.60	0.0036	No	0%
	Woreta	Yes	2.12	0.0297	No	0%
Benishangul-Gumuz	Assosa	Yes	1.47	0.0116	No	0%
	Bambasi	Yes	1.66	0.0013	NA	
	Guba	No	0.69	0.0689	NA	
	Pawe	Yes	1.62	0.0783	NA	
Dire Dawa	Dire Dawa city	Yes	9.04	0.0093	Yes	96%
Gambella	Abobo	Yes	4.02	0.0020	No	0%
	Gambella	Yes	1.60	0.0112	No	0%
Oromia	Adama	Yes	1.64	0.1161	Yes	NA
	Batu	Yes	2.21	0.0328	Yes	77%
	Yabelo	No	0.75	0.0651	NA	
	Jimma Town	Yes	3.82	0.0030	No	0%
	Nekemte Town	Yes	2.73	0.0021	NA	
	Negele Town	Yes	6.04	0.0006	NA	
	Gimbi	Yes	6.31	0.0029	NA	
Southern Nations,	Arba Minch	Yes	1.18	0.1050	Yes	NA

Nationalities and Peoples	Abeshige	Yes	1.32	0.1793	NA	
	Shashago	Yes	1.36	0.0314	NA	
	Hawassa Town	Yes	3.01	0.0303	Yes	62%
Somali	Fik	No	0.69	0.0054	Yes	100%
	Filtu	No	0.70	0.0013	NA	
	Godey	No	0.88	0.1020	Yes	64%
	Degehabur	Yes	1.01	0.4818	Yes	58%
	Kebri Dehar	Yes	1.52	0.0063	Yes	75%
Tigray	Mekele	NA	NA	NA	No	0%
	Tahitay	NA	NA	NA	NA	
	Alamata	Yes #	NA	NA	NA	

† Outbreak status: Outbreak in 2022

‡ Rate ratio: Incidence rate of 2022 over average of 2017–2021

§ Proportion of *An. stephensi*: Combination of larval-reared adults and adult collections. NA: Not available

Outbreak in 2020
